# A Marine Collagen-Based Biomimetic Hydrogel Recapitulates Cancer Stem Cell Niche and Enhances Progression and Chemoresistance in Human Ovarian Cancer

**DOI:** 10.3390/md18100498

**Published:** 2020-09-29

**Authors:** SooHyeon Moon, YeJin Ok, SeonYeong Hwang, Ye Seon Lim, Hye-Yoon Kim, Yong-Jin Na, Sik Yoon

**Affiliations:** 1 Department of Obstetrics and Gynecology, Pusan National University College of Medicine, Yangsan, Gyeongsangnam-do 626-870, Korea; val-kilmer@hanmail.net (S.M.); yjna@pusan.ac.kr (Y.-J.N.); 2 Department of Anatomy, Pusan National University College of Medicine, Yangsan, Gyeongsangnam-do 626-870, Korea; yj0429@pusan.ac.kr (Y.O.); anatomy2017@pusan.ac.kr (S.H.); yeseonlim@pusan.ac.kr (Y.S.L.); solarhy77@naver.com (H.-Y.K.); 3 Immune Reconstitution Research Center of Medical Research Institute, Pusan National University College of Medicine, Yangsan, Gyeongsangnam-do 626-870, Korea

**Keywords:** marine collagen, hydrogel, cancer stem cell, ovarian cancer, 3D cell culture, spheroid, chemoresistance

## Abstract

Recent attention has focused on the development of an effective three-dimensional (3D) cell culture system enabling the rapid enrichment of cancer stem cells (CSCs) that are resistant to therapies and serving as a useful in vitro tumor model that accurately reflects in vivo behaviors of cancer cells. Presently, an effective 3D in vitro model of ovarian cancer (OC) was developed using a marine collagen-based hydrogel. Advantages of the model include simplicity, efficiency, bioactivity, and low cost. Remarkably, OC cells grown in this hydrogel exhibited biochemical and physiological features, including (1) enhanced cell proliferation, migration and invasion, colony formation, and chemoresistance; (2) suppressed apoptosis with altered expression levels of apoptosis-regulating molecules; (3) upregulated expression of crucial multidrug resistance-related genes; (4) accentuated expression of key molecules associated with malignant progression, such as epithelial–mesenchymal transition transcription factors, Notch, and pluripotency biomarkers; and (5) robust enrichment of ovarian CSCs. The findings indicate the potential of our 3D in vitro OC model as an in vitro research platform to study OC and ovarian CSC biology and to screen novel therapies targeting OC and ovarian CSCs.

## 1. Introduction

Ovarian cancer (OC) has the highest death rate among gynecologic cancers and is the fifth leading cause of cancer-related deaths in women, even though it ranks tenth in cancer incidence among women in the United States [1]. According to the American Cancer Society, an estimated 22,240 US were diagnosed as OC, and an estimated 14,070 died from OC in 2018 [2]. The high mortality rate of OC is due to its high incidence rate, diagnosis that is delayed until advanced stages, and the high rate of recurrence despite successful initial therapy [3]. Risk factors that might impact the diverse patterns and trends of OC incidence and mortality include low parity, oral contraceptive use, family history, old age, estrogen/hormone replacement therapy, and changes in diet and physical activity [4].

Chemotherapy with platinum-based agents, such as cisplatin and carboplatin, combined with taxanes such as paclitaxel and docetaxel, is the standard treatment for OC. This first-line chemotherapy often results in a complete response. However, recurrence occurs in 25% of patients with early stage disease and in more than 80% of patients with advanced disease [5]. Accordingly, OC is characterized by the evolution of chemoresistance that presents major clinical and therapeutic challenges to a successful cure. Although the precise mechanisms underlying these characteristic phenomena associated with OC progression and relapse remain unclear, cancer stem cells (CSCs), which can survive first-line chemotherapy, are believed to be responsible, especially for acquired chemoresistance [6,7,8,9,10] and metastasis [11,12,13,14].

CSCs refer to a small group of cells within a cancer that possess stem cell-specific properties, such as self-renewal, maintenance of stemness, proliferation, and the ability to differentiate into multiple cell types. CSCs are critical in the initiation, maintenance, malignant progression, recurrence, metastasis, and drug resistance of cancer [15,16]. Following the discovery of CSCs, many studies have sought to elucidate the characteristics and roles of CSCs to inform potential future therapeutic approaches to overcoming resistance to cancer chemotherapy [17]. In this context, ovarian CSCs could be crucial in the development of effective therapeutic strategies to prevent recurrence and to reduce metastasis in OC. However, while the understanding of the biology of OC and ovarian CSCs has improved, and the therapy of OC has advanced in the past decade, improving the prognosis of patients with relapsed and treatment-resistant disease remains an important challenge [18]. The development of effective combination regimens that eliminate both OC cells and ovarian CSCs could replace conventional therapeutic combination regimens that only target OC cells. This would improve treatment outcomes for OC [19,20,21].

One of the most important steps in establishing ovarian CSC-targeted therapies is to develop adequate enrichment techniques of ovarian CSCs. Various methods have been developed to enrich CSCs. Traditional enrichment methods for CSCs were based on a two-dimensional (2D) monolayer cell culture [22,23]. Techniques have gradually evolved, and scaffold-based three-dimensional (3D) cell culture platforms are becoming increasingly important to enrich and isolate CSCs in many different types of cancer [24,25].

The need for improved understanding of the progression and treatment of cancer has driven the development of more accurate and physiologically relevant in vitro tumor models that serve as important tools in cancer research. These models have enabled the identification of carcinogens and tumor targets, development of cancer therapies and drug screening, and have provided insight into the molecular mechanisms of tumor growth and metastasis [26]. Additionally, the genomic complexity of cancers and the resulting recognition of the value of precision or personalized medicine have driven the adaptation of in vitro tumor models for patient-specific therapeutic management and assessment of metastatic potential. Among the techniques attempted so far, the 3D cell culture, in particular, has enabled the rapid development of new in vitro tumor models that can recapitulate critical steps in tumor biology and metastatic cascades. The expectation is that these models will enable breakthrough innovations in the understanding of chemoresistance, metastasis, oncotargets, biomarkers, drug discovery, and targeted therapies [27].

Biomaterials are critical components of 3D cell culture, as well as tissue engineering and regenerative medicine. These materials are used to construct scaffolds that provide a 3D environment for cell adhesion, migration, differentiation and proliferation, growth, and maintenance by mimicking the natural extracellular matrix (ECM) as structural templates [28,29]. Collagen comprises 25% to 35% of the protein in the human body and is the key structural ECM fibrous protein of biological tissues in invertebrates and vertebrates [30]. Collagen is a valuable biomaterial for diverse applications in various biomedical fields, including tissue engineering, drug delivery, and regenerative medicine, because of its unique properties that include excellent biocompatibility, biodegradability, ready availability, and versatility [31]. Most commercial collagens are obtained from terrestrial mammals, such as cattle and pigs. These collagens are widely used in the food, cosmetic, pharmaceutical, and biomedical industries. However, there are increasing concerns about the use of collagen and collagen-derived products from terrestrial animals. These concerns include purification problems; manufacturing costs; religious dietary rules and restrictions; and the transmission of infectious diseases such as bovine spongiform encephalopathy, transmissible spongiform encephalopathy, and foot-and-mouth disease [32,33].

Marine collagen (MC) derived from marine organisms, such as fish, seaweeds, sponges, and jellyfish, has attracted scientific and industrial interest because of its advantages over mammalian collagen. MC is easily extracted, since it is water-soluble, is safe from livestock diseases, has superior chemical and physical durability, and is abundantly available [34,35,36]. In our previous study, we designed and fabricated a calcium-free, physically crosslinked, transparent, and cost-effective hydrogel matrix composed of MC, alginate, and agarose for use in 3D cell cultures [37]. MC was the key component of the MC-alginate-agarose hydrogel [37]. This MC-based (MC-B) hydrogel exhibited excellent cytocompatibility, generated high yields of multicellular spheroids, and promoted cellular activity [37,38].

Presently, we used the MC-B hydrogel matrix to optimize a bioactive, simple, and efficient novel 3D in vitro human OC model that could efficiently enrich CSCs and enhance malignant properties of human OC cells compared to a 2D culture. The characteristics and efficacy of the 3D in vitro human OC model were evaluated.

## 2. Results

### 2.1. Formation and Growth of OC Cell Spheroids Are Promoted in MC-B Hydrogels

**Figure 1:**
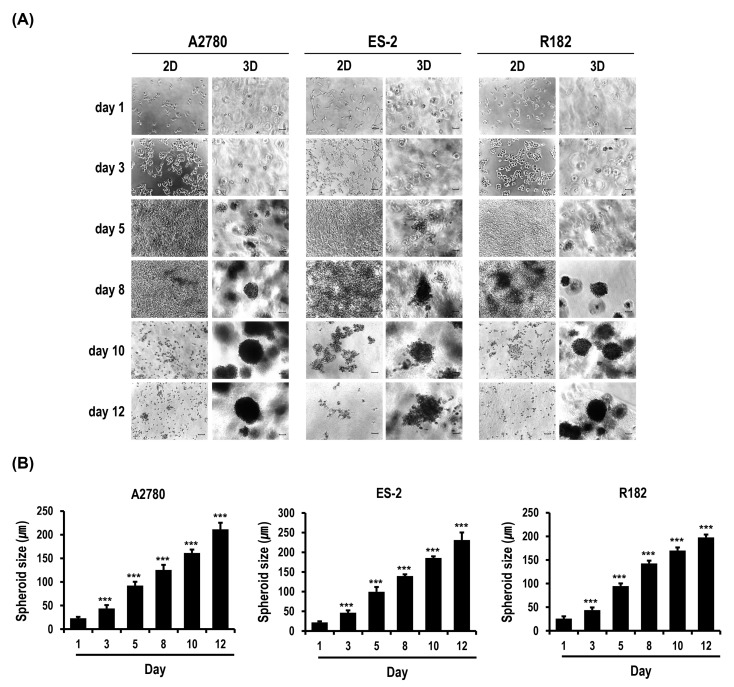
Formation and growth of ovarian cancer (OC) cell spheroids in standard plastic tissue culture plates and marine collagen-based (MC-B) hydrogels. (**A**) Phase contrast microscopy images showing OC cell (A2780, ES-2, and R182 cells) spheroids on culture days 1, 3, 5, 8, 10, and 12 (original magnification ×100). (**B**) Diameters of the OC cell spheroids are depicted in a bar graph. *** *p* < 0.001 vs. those on day 1. Scale bars = 50 μm.

Figure 1A shows phase contrast microscopy images of 2D and 3D OC cells with time. The three cell types (A2780, ES-2, and R182 OC cells) started to form multiple spheroids from day 3. Spheroid numbers gradually increased with time (Figure 1A). Average spheroid diameters measured on day 1, 3, 5, 8, 10, and 12 were 23.0, 43.8, 92.3, 125.4, 161.4, and 211.5 µm, respectively, for A2780 cells, 21.9, 46.4, 99.7, 139.7, 185.6 and 231.3 µm, respectively, for ES-2 cells, and 25.8, 44.8, 94.8, 142.7, 170.0, and 197.9 µm, respectively, for R182 cells (Figure 1B). These spheroids were generally similar in size and shape with time, although the rate of spheroid growth differed slightly depending on the cell type (ES2 > A2780 > R182) (Figure 1A,B). On day 10 of the culture, almost all spheroids from the three cell types were around 200 µm in diameter. This was comparable to a general spheroid-based drug screening size, suggesting that MC-B hydrogels provide a favorable milieu for the growth of OC cell spheroids, which can be applied for the development of diagnostic and therapeutic strategies for OC.

### 2.2. Proliferation and Colony Formation of OC Cells Are Enhanced in MC-B Hydrogels

**Figure 2:**
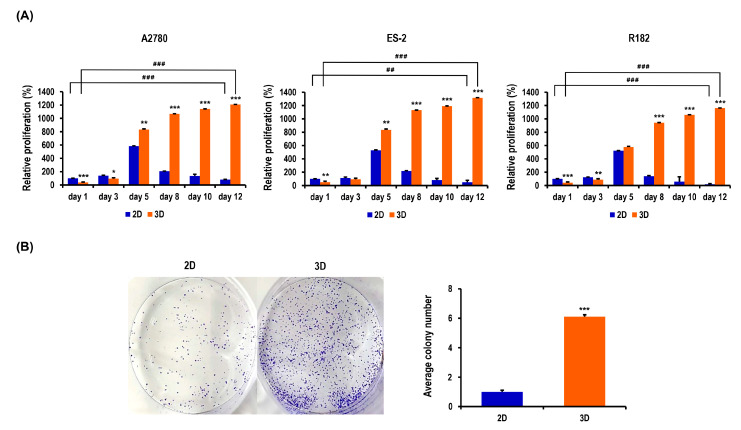
Proliferation and colony formation of OC cells in standard plastic tissue culture plates and MC-B hydrogels. (**A**) Proliferation of A2780, ES-2, and R182 cells on standard plastic tissue culture plates was compared with that in MC-B hydrogels using the WST-1 assay. (**B**) Colony-forming ability of R182 cells cultured under 2D and 3D conditions for 7 days was determined by a colony formation assay. Data represent the means ± SD of three independent experiments. * *p* < 0.05, ** *p* < 0.01, and *** *p* < 0.001 vs. 2D. ^##^ *p* < 0.01 and ^###^ *p* < 0.001 vs. those on day 1.

To quantify the ability of MC-B hydrogels to facilitate cell proliferation, the water-soluble tetrazolium (WST)-1-based colorimetric cell proliferation assay was used. OC cells were successfully propagated in MC-B hydrogels. The cell number in 2D cultures was significantly greater than that in 3D cultures for all three cell types during the first three days of culture (Figure 2A). However, after five days of culture, the numbers in 3D cultures exceeded those in 2D cultures (Figure 2A). On day 5, these cells showed 1.4-fold higher levels of proliferation in 3D than in 2D cultures (*p* < 0.01) for A2780 cells, 1.6-fold (*p* < 0.01) for ES-2 cells, and 1.1-fold for R182 cells. On days 8, 10, and 12, the rates of proliferation in 3D vs. 2D cultures were robustly increased by 5.2-fold (*p* < 0.001), 8.4-fold (*p* < 0.001), and 14.7-fold (*p* < 0.001), respectively, for A2780 cells; 5.2-fold (*p* < 0.001), 14.5-fold (*p* < 0.001), and 26.6-fold (*p* < 0.001), respectively, for ES-2 cells; and 6.7-fold (*p* < 0.001), 18.4-fold (*p* < 0.001), and 63.8-fold (*p* < 0.001), respectively, for R182 cells (Figure 2A). On day 12, 2D cultured cells displayed a significant reduction in number, with values of 0.8-fold (*p* < 0.001) for A2780 cells, 0.5-fold (*p* < 0.01) for ES-2 cells, and 0.2-fold (*p* < 0.001) for R182 cells compared to those on day 1. In contrast, on day 12, 3D cultured cells displayed a dramatic increase in number, with 30.8-fold (*p* < 0.001) for A2780 cells, 26.1-fold (*p* < 0.001) for ES-2 cells, and 27.7-fold (*p* < 0.001) for R182 cells relative to those on day 1 (Figure 2A).

To evaluate the colony-forming abilities of MC-B hydrogels, a clonogenicity assay was performed. At day 10, cells cultured in MC-B hydrogels showed a marked 6.1-fold enhancement in colony formation ability (*p* < 0.001) compared to those in monolayers (Figure 2B). The collective findings indicated that MC-B hydrogels produce environmental conditions that are more suitable for OC cell proliferation and colonization than 2D culture.

### 2.3. Anticancer Drug-Induced Apoptosis of OC Cells Is Suppressed in MC-B Hydrogels

**Figure 3:**
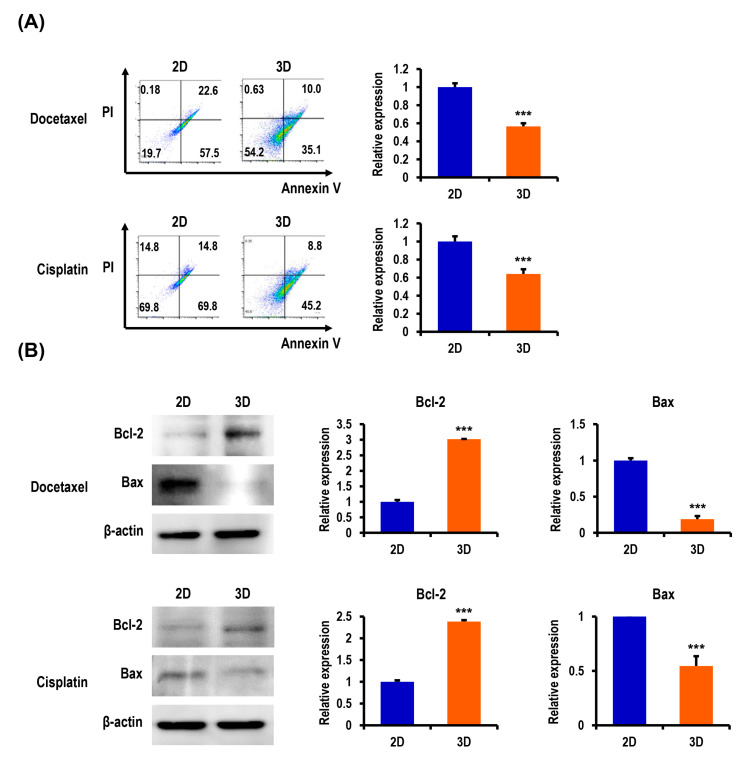
Analysis of the apoptotic state in 3D OC spheroids compared to their 2D cultures. (**A**) Flow cytometric analysis demonstrates an increased resistance to the apoptosis of R182 cells in response to the treatment with 100-nM docetaxel and 12-μM cisplatin (annexin V^+^: apoptotic cells and propidium iodide (PI)^−^: dead cells) in 3D spheroids compared to the 2D culture. The lower left quadrants of the panels (annexin V^−^/PI^−^) represent the intact viable cells, whereas the lower right quadrants (annexin V^+^/PI^−^) represent early apoptotic cells. Apoptosis ratios were calculated from three independent experiments for the cell lines. Bar graphs depict the quantitation of early and late-apoptotic cells (annexin V^+^). (**B**) Western blot analysis of R182 cells for the expression of apoptosis-regulating proteins in 2D and 3D cultures. Antiapoptotic Bcl-2 was upregulated, and proapoptotic Bax was downregulated in 3D cultures as compared with 2D cultures. Bar graphs depict the densitometry quantitation of Bcl-2 and Bax protein expression normalized to β-actin. Data represent the means ± SD of three independent experiments. *** *p* < 0.001 vs. 2D.

Tumor cells grown in 3D models that can more adequately reflect the nature of the in vivo microenvironment are thought to be more resistant to apoptosis induced by antitumor agents than those in traditional 2D cultures. We hypothesized that the multicellular OC spheroids grown within MC-B hydrogels would be less vulnerable to the induction of apoptosis by antitumor agents than cells cultured in 2D. To evaluate the effects of the 3D microenvironment provided by MC-B hydrogels on the susceptibility to apoptosis caused by docetaxel and cisplatin, representative antitumor agents for OC, an annexin V-fluorescein isothiocyanate (FITC) flow cytometry examination was performed. As shown in Figure 3A, a significant difference was evident between 2D and 3D cultures in the degree of apoptosis induction. The rate of viable cells (annexin^−^/ propidium iodide (PI)^−^) and early apoptotic cells (annexin^+^/PI^−^) treated with docetaxel was 54.2% and 35.1%, respectively, for 3D vs. 19.7% and 57.5%, respectively, for 2D. The rate of viable cells (annexin^−^/PI^−^) and early apoptotic cells (annexin^+^/PI^−^) treated with cisplatin was 45.6% and 45.2%, respectively, for 3D vs. 15.3% and 69.8%, respectively, for 2D. The findings indicated that the resistance to apoptosis induced by docetaxel and cisplatin was enhanced in cells cultured in 3D compared to 2D.

To determine the mechanism employed in the regulation of apoptosis in the 3D multicellular spheroids, we further examined the expression of apoptosis-related proteins by Western blot analysis. The treatment of 3D-cultured R182 cells with docetaxel and cisplatin upregulated the expression of an antiapoptotic protein Bcl-2 by 3.0-fold (*p* < 0.001) and 2.4-fold (*p* < 0.001), respectively, and suppressed the expression of a proapoptotic protein Bax by 0.2-fold (*p* < 0.001) and 0.5-fold (*p* < 0.001), respectively, compared to the treatment of 2D controls (Figure 3B). The findings indicated that the activation of the Bcl-2 family proteins (Bcl-2 and Bax), which are the key regulators of apoptosis, is involved in the molecular mechanism by which the 3D microenvironment in MC-B hydrogels regulates apoptosis in OC cells.

### 2.4. Metastatic Potentials of OC Cells Are Elevated in MC-B Hydrogels

**Figure 4:**
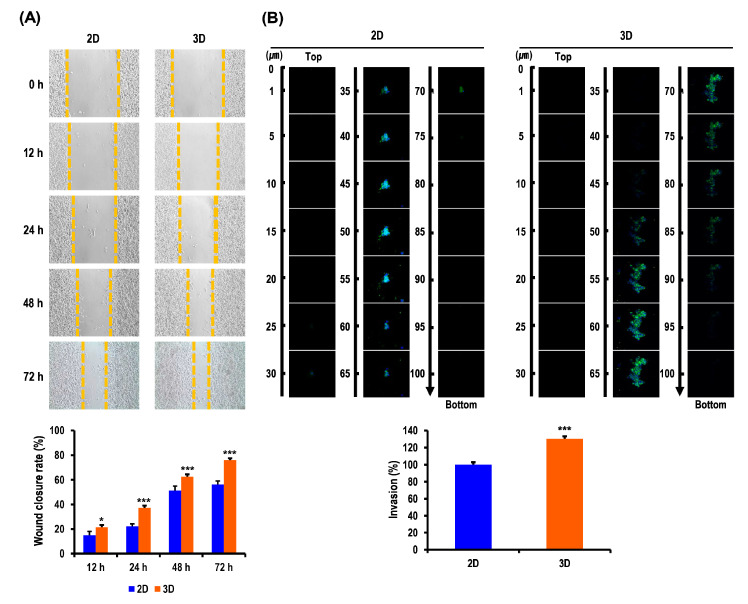
Migratory and invasive behavior of OC cells in standard plastic tissue culture plates and MC-B hydrogels. (**A**) Representative phase contrast microscopy images from a wound-healing assay of R182 cells migrating into the cell-free space. Images were captured at 0, 12, 24, 48, and 72 h. The distances between the two edges were scaled for three positions at different times. The 3D MC-B scaffold culture environment enhanced the migration ability of R182 cells compared with the 2D plate culture environment. (**B**) The invasive potential of 2D- and 3D-cultured A2780 cells in a hydrogel invasion assay. The 3D MC-B-scaffold culture environment enhanced the invasive ability of A2780 cells compared with the 2D plate culture environment. Data represent the mean ± SD of three independent experiments. * *p* < 0.05 and *** *p* < 0.001 vs. 2D.

Metastasis is a multistep process that includes the migration and invasion of cancer cells, which are hallmarks of cancer metastasis. Tumor cells grown in 3D models that can more accurately recapitulate the enormous complexity of in vivo biological systems are considered to exhibit a higher level of metastatic potentials over those in a traditional 2D monolayer. Thus, we speculated that the 3D multicellular OC spheroids would exhibit enhanced metastatic potential compared to cells cultured in 2D. To assess this, wound-healing and invasion assays were performed on A2780 cells. Images of scratch areas in the wound-healing assay from the time points 0, 12, 24, 48, and 72 h are presented in Figure 4A. At 12, 24, 48, and 72 h, the rate of wound closure in the 3D cultures was 21.6% (vs. 4.8% for 2D, *p* < 0.05), 37.3% (vs. 22.3% for 2D, *p* < 0.001), 62.7% (vs. 51.4% for 2D, *p* < 0.001), and 76.0% (vs. 56.1% for 2D, *p* < 0.001), respectively (Figure 4A). We studied the invasion of A2780 cells by measuring the net invasion depth of the cells into the hydrogel. The hydrogel invasion assay demonstrated that the 3D culture exhibited a significantly higher rate of cell invasion (130.2%, *p* < 0.001) in comparison to the 2D culture (Figure 4B). Collectively, these data provided evidence that the 3D niche provided by MC-B hydrogels facilitates the migration and invasion of OC cells and promotes their metastatic potential.

### 2.5. Chemoresistance of OC Cells Is Increased in MC-B Hydrogels

**Figure 5:**
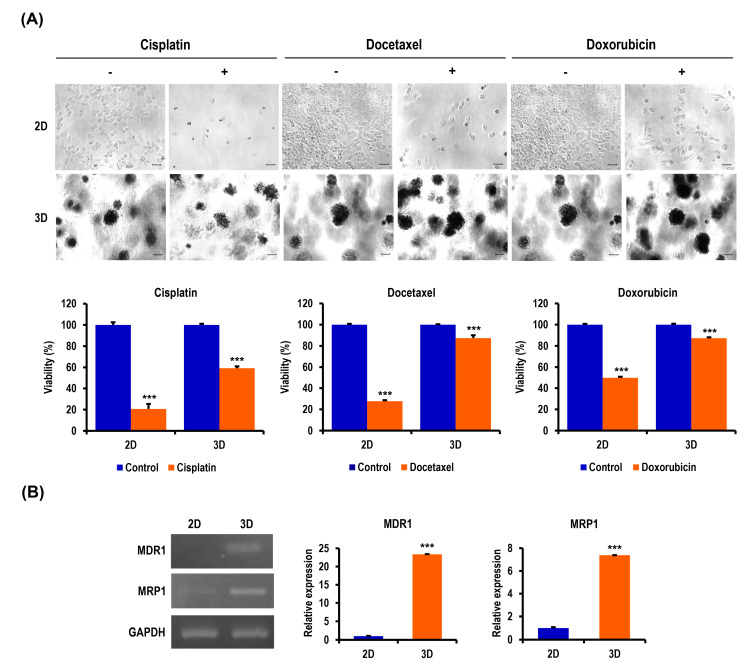
Sensitivity to chemotherapeutics and the expression of multidrug resistance-related genes in 2D and 3D OC cell cultures. (**A**) Phase contrast microscopy images (original magnification ×100) and cell viabilities of R182 cells in 2D and 3D cultures after exposure to cisplatin, docetaxel, and doxorubicin. Data represent the mean percentage viability ± SD of three independent experiments normalized against untreated control cells. *** *p* < 0.001 vs. 2D. (**B**) Reverse transcription (RT)-PCR demonstrates the upregulated expression of multidrug resistance 1 (MDR1) and multidrug resistance-associated protein 1 (MRP1) genes important in promoting malignancy and multidrug resistance in 3D spheroids compared to the 2D culture. Bar graphs depict the densitometry quantitation of MDR1 and MRP1 mRNA expression normalized to glyceraldehyde-3-phosphate dehydrogenase (GAPDH) mRNA. Data represent the means ± SD of three independent experiments. *** *p* < 0.001 vs. 2D.

Multidrug resistance remains a major obstacle to successful cancer chemotherapy. Tumor cells grown in 3D models that can more closely mimic the properties of living tissues exhibit a higher level of drug resistance over those in traditional 2D monolayers. Thus, we speculated that the 3D multicellular OC spheroids grown within MC-B hydrogels would display enhanced chemotherapeutic resistance to antitumor agents for OC compared to cells cultured in 2D. To evaluate the effects of the 3D microenvironment provided by MC-B hydrogels on drug resistance against various antitumor agents for OC, cellular cytotoxicity and morphology were assessed. The WST-1-based colorimetric cell viability assay revealed a significant difference between 2D and 3D cultures concerning antitumor agent sensitivity. Three-dimensional cultured cells treated with 12-μM cisplatin, 100-nM docetaxel, and 1-μM doxorubicin for 24 h displayed 2.9-fold (*p* < 0.001), 3.2-fold (*p* < 0.001), and 1.7-fold (*p* < 0.001) increases in chemoresistance, respectively, compared to those in 2D-cultured cells (Figure 5A). Untreated cells grown for the same length of time served as the control (considered as 100%). These cytotoxicity results were also concomitant with the morphological changes observed by phase contrast microscopy (Figure 5B). These data demonstrated that the antitumor agent resistance of OC cells was markedly augmented in 3D cultures compared to 2D cultures.

To investigate the effects of the 3D niche provided by MC-B hydrogels on the expression of multidrug resistance-related genes, which play a critical role in the acquisition of chemoresistance in multiple cancers, cells grown in 2D or 3D cultures were examined. The genes examined encoded multidrug resistance 1 (MDR1) and multidrug resistance-associated protein 1 (MRP1). MDR1, which is also termed the permeability glycoprotein (P-glycoprotein, P-gp), or ATP-binding cassette subfamily B member 1 (ABCB1). MRP1 is also termed ABCC1. The expression of the MDR1 and MRP1 genes was highly upregulated by 23.4-fold (*p* < 0.001) and 7.4-fold (*p* < 0.001), respectively, in 3D-cultured A2780 cells compared to the 2D controls (Figure 5B). These results suggested that drug resistance in cells grown in 3D MC-B hydrogel scaffolds could be attributed to an increased drug efflux.

### 2.6. Ovarian CSC Biomarker Expression Is Augmented in MC-B Hydrogels

Flow cytometry was used to assess the CSC biomarker expressions in OC cells cultured in 2D and 3D conditions. The biomarkers of CSCs in OC included CD44, CD117, and CD133. The expression of typical cell surface phenotypes such as CD44^+^, CD117^+^, CD133^+^, CD44^+^/CD117^+^, CD44^+^/CD133^+^, and CD117^+^/CD133^+^ was selected to detect the CSC proportions in 2D and 3D human OC cells (A2780, ES-2, and R182 cells).

**Figure 6:**
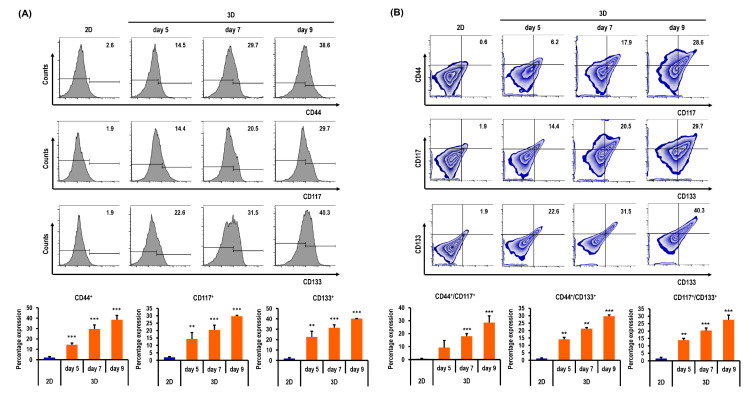
Quantitative flow cytometry analyses of A2780 cells in standard plastic tissue culture plates and MC-B hydrogels. (**A**) Cells grown in MC-B hydrogels display higher CD44, CD117, and CD133 expression than those cultured in the conventional 2D culture method on days 5, 7, and 9. (**B**) Double-positive expression patterns of cancer stem cell (CSC) markers CD44^+^/CD117^+^, CD44^+^/CD133^+^, and CD117^+^/CD133^+^ were compared between 2D and 3D cell cultures. Cells grown in MC-B hydrogels display higher CD44^+^/CD117^+^, CD44^+^/CD133^+^, and CD117^+^/CD133^+^ expressions than those cultured in the conventional 2D culture method on days 5, 7, and 9. Data represent the means ± SD of three independent experiments. ** *p* < 0.01 and *** *p* < 0.001 vs. 2D.

Figure 6A shows the flow cytometry data of A2780 cells in 2D on day 3 and in spheroids on days 5, 7, and 9. The percentages of CD44^+^ A2780 cells were significantly higher in 3D on days 5, 7, and 9 by 6.9-fold (*p* < 0.001), 14.1-fold (*p* < 0.001), and 18.4-fold (*p* < 0.001), respectively, than in 2D (Figure 6A). The percentages of CD117^+^ A2780 cells were also significantly higher in 3D on days 5, 7, and 9 by 7.6-fold (*p* < 0.01), 10.8-fold (*p* < 0.001), and 15.6-fold (*p* < 0.001), respectively, than in 2D (Figure 6A). The percentages of CD133^+^ A2780 cells were significantly higher in 3D on days 5, 7, and 9 by 11.9-fold (*p* < 0.01), 16.6-fold (*p* < 0.001), and 21.2-fold (*p* < 0.001), respectively, than in 2D (Figure. 6A). The percentages of both CD44^+^/CD117^+^ A2780 cells were significantly higher in 3D on days 5, 7, and 9 by 15.3-fold (*p* > 0.05), 30.0-fold (*p* < 0.001), and 47.7-fold (*p* < 0.001), respectively, than in 2D (Figure 6B). The percentages of both CD44^+^/CD133^+^ A2780 cells were significantly higher in 3D on days 5, 7, and 9 by 11.7-fold (*p* < 0.01), 17.8-fold (*p* < 0.01), and 24.8-fold (*p* < 0.001), respectively, than in 2D (Figure 6B). The percentages of both CD117^+^/CD133^+^ A2780 cells were significantly higher in 3D on days 5, 7, and 9 by 9.2-fold (*p* < 0.01), 13.6-fold (*p* < 0.001), and 18.4-fold (*p* < 0.001), respectively, than in 2D (Figure 6B).

**Figure 7:**
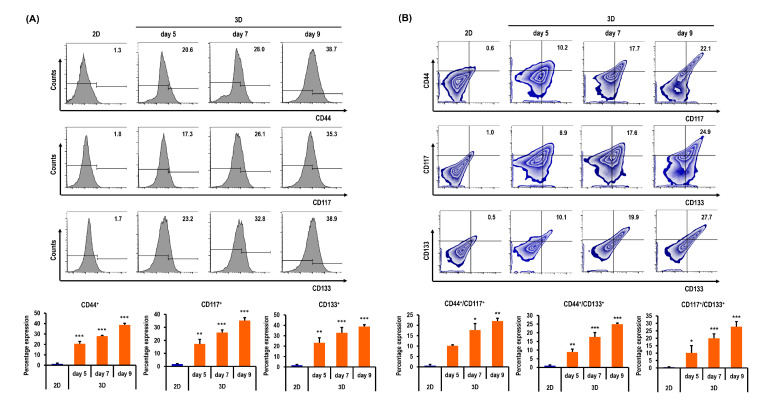
Quantitative flow cytometry analyses of ES-2 cells in standard plastic tissue culture plates and MC-B hydrogels. (**A**) Cells grown in MC-B hydrogels display higher CD44, CD117, and CD133 expressions than those cultured in the conventional 2D culture method on days 5, 7, and 9. (**B**) Double-positive expression patterns of CSC markers CD44^+^/CD117^+^, CD44^+^/CD133^+^, and CD117^+^/CD133^+^ were compared between 2D and 3D cell cultures. Cells grown in MC-B hydrogels display higher CD44^+^/CD117^+^, CD44^+^/CD133^+^, and CD117^+^/CD133^+^ expressions than those cultured in the conventional 2D culture method on days 5, 7, and 9. Data represent the means ± SD of three independent experiments. * *p* < 0.05, ** *p* < 0.01, and *** *p* < 0.001 vs. 2D.

Figure 7A shows the flow cytometry data of ES-2 cells in 2D on day 3 and in spheroids on days 5, 7, and 9. The percentages of CD44^+^ ES-2 cells were significantly higher in 3D on days 5, 7, and 9 by 14.8-fold (*p* < 0.001), 20.0-fold (*p* < 0.001), and 27.7-fold (*p* < 0.001), respectively, than in 2D (Figure 7A). The percentages of CD117^+^ ES-2 cells were significantly higher in 3D on days 5, 7, and 9 by 9.6-fold (*p* < 0.01), 14.5-fold (*p* < 0.001), and 19.6-fold (*p* < 0.001), respectively, than in 2D (Figure 7A). The percentages of CD133^+^ ES-2 cells were significantly higher in 3D on days 5, 7, and 9 by 13.6-fold (*p* < 0.01), 19.3-fold (*p* < 0.001), and 22.9-fold (*p* < 0.001), respectively, than in 2D (Figure 7A). The percentages of both CD44^+^/CD117^+^ ES-2 cells were significantly higher in 3D on days 5, 7, and 9 by 16.5-fold (*p* > 0.05), 28.5-fold (*p* < 0.05), and 35.6-fold (*p* < 0.01), respectively, than in 2D (Figure 7B). The percentages of both CD44^+^/CD133^+^ ES-2 cells were significantly higher in 3D on days 5, 7, and 9 by 9.0-fold (*p* < 0.01), 17.6-fold (*p* < 0.001), and 25.0-fold (*p* < 0.001), respectively, than in 2D (Figure 7B). The percentages of both CD117^+^/CD133^+^ ES-2 cells were significantly higher in 3D on days 5, 7, and 9 by 20.2-fold (*p* < 0.05), 39.8-fold (*p* < 0.001), and 55.6-fold (*p* < 0.001), respectively, than in 2D (Figure 7B).

**Figure 8:**
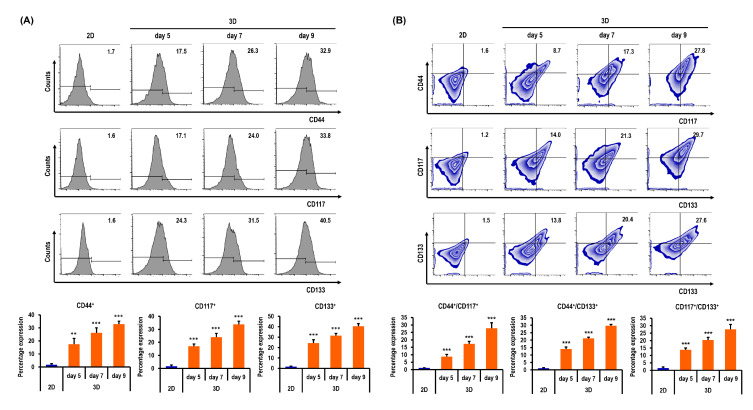
Quantitative flow cytometry analyses of R182 cells in standard plastic tissue culture plates and MC-B hydrogels. (**A**) Cells grown in MC-B hydrogels display higher CD44, CD117, and CD133 expressions than those cultured in the conventional 2D culture method on days 5, 7, and 9. (**B**) Double-positive expression patterns of CSC markers CD44^+^/CD117^+^, CD44^+^/CD133^+^, and CD117^+^/CD133^+^ were compared between 2D and 3D cell cultures. Cells grown in MC-B hydrogels show higher CD44^+^/CD117^+^, CD44^+^/CD133^+^, and CD117^+^/CD133^+^ expressions than those cultured in the conventional 2D culture method on days 5, 7, and 9. Data represent the means ± SD of three independent experiments. ** *p* < 0.01 and *** *p* < 0.001 vs. 2D.

Figure 8A shows the flow cytometry data of R182 cells in 2D on day 3 and in spheroids on days 5, 7, and 9. The percentages of CD44^+^ R182 cells were significantly higher in 3D on days 5, 7, and 9 by 10.2-fold (*p* < 0.01), 15.3-fold (*p* < 0.001), and 19.1-fold (*p* < 0.001), respectively, than in 2D (Figure 8A). The percentages of CD117^+^ R182 cells were significantly higher in 3D on days 5, 7, and 9 by 10.7-fold (*p* < 0.001), 15.0-fold (*p* < 0.001), and 21.1-fold (*p* < 0.001), respectively, than in 2D (Figure 8A). The percentages of CD133^+^ R182 cells were significantly higher in 3D on days 5, 7, and 9 by 15.2-fold (*p* < 0.001), 19.7-fold (*p* < 0.001), and 25.3-fold (*p* < 0.001), respectively, than in 2D (Figure 8A). The percentages of both CD44^+^/CD117^+^ R182 cells were significantly higher in 3D on days 5, 7, and 9 by 7.9-fold  (*p* < 0.001), 15.7-fold (*p* < 0.001), and 25.3-fold (*p* < 0.001), respectively, than in 2D (Figure 8B). The percentages of both CD44^+^/CD133^+^ R182 cells were significantly higher in 3D on days 5, 7, and 9 by 11.7-fold (*p* < 0.001), 17.8-fold (*p* < 0.001), and 24.8-fold (*p* < 0.001), respectively, than in 2D (Figure 8B). The percentages of both CD117^+^/CD133^+^ R182 cells were significantly higher in 3D on days 5, 7, and 9 by 9.2-fold (*p* < 0.001), 13.6-fold (*p* < 0.001), and 18.4-fold (*p* < 0.001), respectively, than in 2D (Figure 8B).

### 2.7. Stemness and Pluripotency Marker Expression of OC Cells Is Enhanced in MC-B Hydrogels

**Figure 9:**
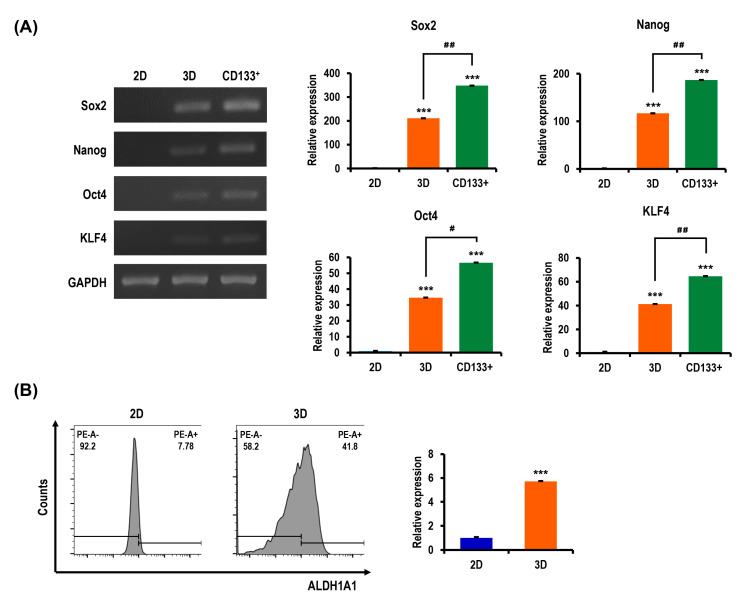
Expression of stemness and pluripotency biomarkers in R182 cell spheroids compared to 2D cultures. (**A**) RT-PCR gene expression analysis of stemness and pluripotency regulatory factors (Sox2, Nanog, Oct4, and KLF4) in 2D-cultured cells, 3D spheroids, and the isolated CD133^+^ CSCs from 3D spheroids. Bar graphs show the relative expressions of genes in cells grown in these cells. GAPDH was used as a housekeeping gene for RT-PCR data normalization. Results are expressed as mean ± SD (*n* = 3). (**B**) Flow cytometry comparative analysis of the expression of aldehyde dehydrogenase 1 family member A1 (ALDH1A1), a functional stem cell marker, in 2D and 3D cultures. Data represent the means ± SD of three independent experiments. *** *p* < 0.001 vs. 2D. ^#^ *p* < 0.05 and ^##^ *p* < 0.01 vs. 3D spheroids.

Similar to the flow cytometry examination of ovarian CSC biomarker expression, we examined the expression of key genes associated with cancer stemness and pluripotency to confirm the CSC enrichment efficiency of our 3D OC cell culture model. The expressions of crucial regulators of stemness and pluripotency in stem cells (Sox2, Oct4, Nanog, and KLF4) were robustly augmented in 3D cultures compared to 2D cultures (Figure 9A). In 3D culture, the rates of Sox2, Nanog, Oct4, and KLF4 expressions were increased 210.9-fold  (*p* < 0.001), 116.8-fold  (*p* < 0.001), 34.6-fold  (*p* < 0.001), and 41.2-fold  (*p* < 0.001), respectively, compared with those in the 2D culture (Figure 9A). Notably, in isolated CD133^+^ CSCs from the 3D culture, the rates of Sox2, Nanog, Oct4, and KLF4 expressions were increased by 348.0-fold  (*p* < 0.001), 186.6-fold  (*p* < 0.001), 56.6-fold  (*p* < 0.001), and 64.6-fold  (*p* < 0.001), respectively, compared with those in the 2D culture (Figure 9A). In isolated CD133^+^ CSCs from the 3D culture, the rates of Sox2, Nanog, Oct4, and KLF4 expressions were increased 1.6-fold  (*p* < 0.01), 1.6-fold  (*p* < 0.01), 1.6-fold  (*p* < 0.05), and 1.6-fold  (*p* < 0.01), respectively, compared with those in the 3D culture (Figure 9A). A flow cytometry analysis of cells cultured in 3D revealed increased levels of aldehyde dehydrogenase 1 family member A1 (ALDH1A1), a functional stem cell marker (5.7-fold, *p* < 0.001) compared to 2D-cultured cells (Figure 9B). The collective results indicated the value of MC-B hydrogels for the efficient enrichment of CSCs.

### 2.8. Aggressiveness of OC Cells Is Reinforced in MC-B Hydrogels

Tumor cells grown in 3D models that can efficiently reproduce the characteristics of the in vivo tissue milieu are believed to have more malignant phenotypes than those in traditional 2D monolayer cultures. Thus, we speculated that the multicellular 3D OC spheroids generated in MC-B hydrogels would display expedited progression compared to cells cultured in 2D. We evaluated the expression of the molecules that are essential for tumor aggressiveness, to explore the molecular mechanism by which the 3D niche provided by MC-B hydrogels regulates malignant cell behavior and tumor progression in OC.

**Figure 10:**
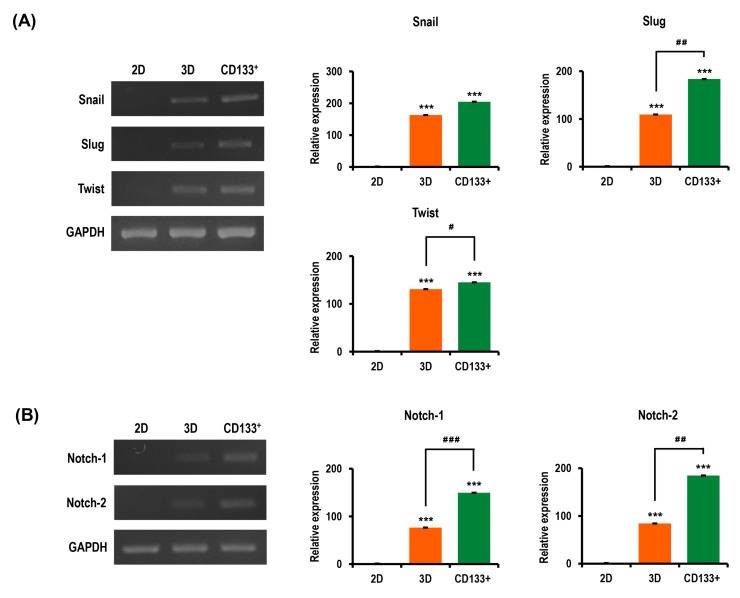
Expression of molecules associated with cancer aggressiveness in R182 cell spheroids compared to 2D cultures. (**A**) RT-PCR gene expression analysis of epithelial–mesenchymal transition (EMT)-driving factors (Snail, Slug, and Twist) in 2D-cultured cells, 3D spheroids, and the isolated CD133^+^ CSCs from 3D spheroids. Bar graphs show relative expressions of genes in cells grown in these cells. GAPDH was used as a housekeeping gene for RT-PCR data normalization. Results show mean ± SD (*n* = 3). (**B**) RT-PCR demonstrates the upregulated gene expressions of Notch-1 and -2, which are important in tumor promotion and progression in 3D spheroids compared to the 2D culture. Bar graphs plot the densitometry quantitation of Notch-1 and -2 mRNA expressions normalized to GAPDH mRNA. Data represent the means ± SD of three independent experiments. *** *p* < 0.001 vs. 2D. ^#^ *p* < 0.05, ^##^ *p* < 0.01, and ^###^ *p* < 0.001 vs. 3D spheroids.

Multiple lines of evidence have established that epithelial–mesenchymal transition (EMT) is vital in tumor growth, progression, invasion, dissemination, metastasis, and drug resistance [39,40]. The three-dimensional culture in MC-B hydrogels strikingly upregulated the expression of the pivotal EMT transcription factors Snail, Slug, and Twist by 163.0-fold (*p* < 0.001), 109.3-fold (*p* < 0.001), and 130.9-fold (*p* < 0.001), respectively, compared with that of the 2D culture (Figure 10A). Furthermore, in isolated CD133^+^ CSCs from 3D cultures, the expression levels of Snail, Slug, and Twist were 204.8-fold  (*p* < 0.001), 183.7-fold  (*p* < 0.001), and 145.2-fold  (*p* < 0.001) higher, respectively, compared with those in the 2D culture (Figure 10A). Of interest, in isolated CD133^+^ CSCs from the 3D culture, the expression levels of Snail, Slug, and Twist were 1.3-fold (*p* < 0.05), 1.7-fold (*p* < 0.01), and 1.1-fold (*p* < 0.05) higher than those of the respective values observed in the 3D culture (Figure 10A).

Activation of the Notch signaling pathway is important in the proliferation and progression of a variety of tumor cell types, including OC, as well as in CSC maintenance [21]. Thus, we evaluated the expressions of Notch-1 and Notch-2 to further explore the molecular mechanism by which the 3D microenvironment in MC-B hydrogels regulates tumor aggressiveness in OC. As shown in Figure 10B, in the 3D culture, the levels of Notch-1 and Notch-2 expression were increased 76.3-fold (*p* < 0.001) and 84.3-fold (*p* < 0.001), respectively, compared with those in the 2D culture (Figure 10A). Notably, in isolated CD133^+^ CSCs obtained from 3D cultures, the levels of Notch-1 and Notch-2 expression were 149.6-fold and 184.7-fold higher, respectively, compared with those in the 2D culture (Figure 10B). In isolated CD133^+^ CSCs from the 3D cultures, the levels of Notch-1 and Notch-2 expression were increased by 2.0-fold (*p* < 0.001) and 2.2-fold (*p* < 0.01), respectively, compared with those in the 3D culture (Figure 10B).

## 3. Discussion

The tumor microenvironment is formed by complex tissue containing various components, such as the ECM and a signaling network of cytokines, chemokines, growth factors, hormones, cell adhesion molecules, and matrix metalloproteinases that control cell-cell communications and cell-ECM interactions. This microenvironment plays a key role in facilitating tumor progression, including cell survival, adhesion, proliferation, differentiation, migration, invasion, the metastasis recurrence of tumor cells, and the acquisition of resistance to chemoradiotherapy [41,42]. Three-dimensional culture systems can more closely replicate a variety of critical biological phenomena observed in in vivo tissue, such as gene and protein expressions and cell survival, proliferation, adhesion, migration, development, and differentiation, in both the functional and morphological aspects [27,43]. Accordingly, the 3D cell culture has quickly emerged as one of the most promising platforms for a suitable in vitro tumor model to recapitulate the in vivo behavior of tumor cells. This platform can substitute for a conventional 2D cell culture, which has many limitations and is a poor surrogate of the complex 3D natural tissue milieu.

OC is the most lethal gynecological malignancy, despite its responsiveness to therapy. The high failure rate associated with drug development specific for OC may be linked to the lack of reliable preclinical in vitro testing models [44,45,46]. Many mouse models have been developed to evaluate the various features of OC in humans to shed light on tumorigenesis, tumor progression, and therapeutic strategies [47,48,49]. However, animal models have significant limitations—particularly, cost, time, ethical concerns, and poor relevance to human biology. In this light, the 3D in vitro OC cell culture is increasingly being recognized as a pivotal tool for the better understanding of the biological mechanisms underlying cancer progression, such as drug resistance and metastasis, to identify biomarkers and to develop therapeutic strategies in OC [50]. Although considerable research has been devoted to 3D in vitro cell culture models for OC to capture cancer complexity in vitro, no definitive and ideal model has yet been established, and detailed studies still continue to provide exciting new information.

Multidrug resistance is a complex biological phenomenon in which cancer cells develop a resistance to a variety of structurally unrelated anticancer drugs. This resistance is the main cause of disease relapse and death in cancer patients [51]. Chemotherapy resistance can be mediated by a number of different mechanisms, which involve the increased efflux of drugs from cells, which is mediated by membrane transporter proteins, such as MDR1 (P-gp) and MRP1. The precise mechanism is unclear [52,53]. The ATP-binding cassette (ABC) superfamily of transporter proteins, including MDR1 and MRP1, utilize the energy released from ATP hydrolysis to pump out cytotoxic drugs from cancer cells, leading to a limited exposure to chemotherapeutic drugs [54]. In addition, resistance can result from defective apoptotic pathways or changes in the cell cycle mechanisms due to malignant transformation and/or exposure to chemotherapy [55].

Consistent with these facts, we observed that the anticancer drug-induced apoptosis of OC cells was suppressed, and the chemoresistance of OC cells against antitumor agents was increased, along with the upregulated expression of major drug resistance genes MDR1 (P-gp) and MRP1 in 3D MC-B hydrogels. These findings demonstrated that our OC model efficiently mimics the real tumor microenvironment. Additionally, the average spheroid sizes of the A2780, ES-2, and R182 OC cells on day 10 were approximately 200 µm, which is the optimal size commonly used for spheroid-based drug screening [56,57,58]. Moreover, multicellular spheroids with a diameter larger than approximately 100 μm contain an internal hypoxic zone caused by the limited distribution of oxygen, nutrients, and metabolites and a necrotic core [59,60]. The hypoxic tumor microenvironment is considered a critical component in determining drug resistance through specific cellular signaling pathways and plays a vital role in regulating the resistance of CSCs to chemotherapy and radiotherapy [61,62,63,64,65]. These findings suggest that our MC-B hydrogel provides a suitable milieu for the growth of these OC cell spheroids, which can be applied for the development of diagnostic and therapeutic strategies for OC.

Additionally, and importantly, we established an efficient CSC enrichment method with our 3D spheroid cell culture using MC-B hydrogels. Our data revealed essentially similar efficiencies of CSC enrichment among the A2780, ES2, and R182 OC cells, as well as different ovarian CSC biomarkers (CD44^+^, CD117^+^, CD133^+^, CD44^+^/CD117^+^, CD44^+^/CD133^+^, and CD117^+^/CD133^+^), although there was a slight difference in the levels of CSC generation depending on types of cells, as well as the types of ovarian CSC biomarkers. It must also be stressed that, despite the continued debate on the existence of CSCs, a growing body of evidence indicates that they are the main source of chemoradioresistance and are thought to be the major cause of cancer therapy failure [7,21,66,67,68,69]. Furthermore, CSCs have been proposed to be the key cells that have escaped chemotherapy by the development of an acquired drug resistance following chemotherapeutic treatments or irradiation. These CSCs serve as the central tumor-initiating cells during recurrence and metastasis, possibly due to their high DNA repair capacity, expression of multidrug resistance, and ability to self-renew and differentiate into heterogeneous lineages of cancer cells [8,70,71]. Based on the role of CSCs in drug resistance and cancer relapse, and their therapeutic implications and perspectives in cancer therapy, there is an urgent need to advance our understanding of the function of CSCs in cancer initiation and progression and, more importantly, develop novel CSC-specific targeting strategies, especially for the treatment of multidrug-resistant and metastatic tumors [72]. In particular, much attention has been dedicated to CSC research, especially to designing approaches to induce cell death in CSCs. The aim is to develop effective therapeutics to result in cancer regression and to avoid cancer relapse after therapy. Although progress has been made through the development of therapeutic strategies to target CSCs, their therapeutic efficacy remains insufficient due to the absence of specific targetable biomarkers [72]. Thus, the development of innovative CSC-specific targeting strategies will offer great hope for future effective therapies for multidrug-resistant, metastatic, and recurrent OC. To this end, one of the most important approaches is to establish a method to effectively and economically enrich CSCs.

In the last decade, various strategies based on conventional 2D cell culture platforms have been implemented to develop methods for CSC enrichment, including density gradient centrifugation [73]; hypoxia culture [22,74]; chemoradiotherapy stimulation [23,75,76,77]; side population sorting [78,79]; mesenchymal stem cell secretome cultures [80,81]; and molecule-mediated triggering, such as neural stimulating factor [82], estrogen [83], progestin [84], hypoxia-inducible factor (HIF)-1 [85], E-cadherin [86], keratin [87], poly ADP ribose polymerase (PARP) inhibitors [88], and hepatocyte growth factor (HGF)/mesenchymal-epithelial transition (c-Met) [89]. Overall, however, 2D culture conditions provide limited expansion, and the cells tend to lose clonal and differentiation capacity upon long-term passaging. The two-dimensional culture also lacks the intricacy necessary to mimic the CSC niche, dynamic, and specialized 3D microenvironments, which are responsible for the regulation of CSC fate in vivo [90].

Whereas these efforts have contributed significantly to the conception and design of CSC enrichment methods, there is currently intense interest in 3D cell cultures for more effective and efficient methods of CSC enrichment to overcome some of the limitations of the 2D-based approaches. A variety of strategies based on a 3D cell culture platform for CSC enrichment, which can be classified as non-scaffold-based or scaffold-based techniques, have shown the benefits and advantages in the enrichment and characterization of CSCs in vitro more than those based on 2D culture.

First, scaffold-free techniques are cell aggregate-based, including hanging drops, nonadherent or ultra-low attachment plates, and magnetic biolevitation, and rely on physical forces to bring the cells together and cell-cell adhesions to form the aggregate [91,92,93,94,95,96,97,98,99]. In particular, the nonadherent or ultra-low attachment cell culture, also termed the sphere formation assay, forms cell spheroids that are free-floating in the liquid culture media with a serum-free medium containing growth factors and has been widely used for the culture of tumorspheres [92,95,96,98]. This liquid culture without a gel matrix on nonadherent or ultra-low attachment plates can be practically handled as a clear liquid. However, cell aggregates (spheres or spheroids) generated in the medium often form large clumps exceeding 500 μm in diameter due to the uncontrollable spontaneous fusion among spheres and adherent sphere growth. The results are a slow cell proliferation rate and poor diffusion of nutrients. These large spheroids may lead to pseudo-resistance to anticancer drugs [100,101,102]. Moreover, numerous challenges remain with the traditional suspension culture system. These include low yield and difficulty in maintaining spheroids and changing the media procedure [90,91,103].

Second, different scaffold-based 3D cell culture techniques that use various types of scaffolds have recently been reported to develop advanced methods for CSC enrichment. These include chitosan-alginate scaffolds [104], polyelectrolyte multilayer films [105], hyaluronic acid-based multilayer films [106], porous chitosan-alginate scaffolds [107], poly (ε-caprolactone) scaffolds [108], Matrigel^®^ [109], agarose multi-well dishes [110], methylcellulose and gellan gum [103], and hyaluronic acid hydrogels [111]. Despite recent advancements in CSC-enrichment techniques, improving the methods for enrichment of the desired CSCs remains an important challenge.

The Notch pathway is one of the most intensively studied candidate therapeutic targets for tumor cells, as Notch signaling is critical for cell proliferation, aggressiveness, and chemoresistance, as well as stem cell propagation in diverse types of primary and metastatic tumors [112]. The molecular mechanisms underlying the acquisition of chemoresistance coordinated by Notch signaling are believed to involve the induction of the EMT, the formation of tumor stem cells, and the upregulated expression of MDR such as the MDR1 and MRP1 [21,113,114,115]. EMT is associated with metastasis, as well as with the generation and maintenance of CSCs, contributing to tumor invasion, heterogeneity, and chemoresistance [39,40]. Thus, many researchers are striving to develop targeted therapeutic strategies to inhibit the Notch pathway, EMT-driving transcription factors, and cancer stemness-related molecules for the successful treatment of human malignancies.

Intriguingly, we observed that our MC-B hydrogels provide a favorable milieu for several aspects. The first was the survival, proliferation, colony formation, migration, invasion, and CSC formation of OC cells. The second was the significant enrichment of ovarian CSCs, representing up to 55.6-fold enrichment over the 2D monolayer culture. The third was the acquisition of an enhanced malignant phenotype, such as acquired chemoresistance, metastatic potential, and stemness in OC cells, which can be used for the development of diagnostic, treatment, and preventive strategies against OC. In the present study, we describe a novel, simple, rapid, and efficient approach not only to enrich ovarian CSCs in terms of generation time and yield of CSCs but, also, to engineer a 3D in vitro model of OC chemoresistance and aggressiveness, with a special interest in ovarian CSCs. This model more closely recapitulates the in vivo microenvironment than traditional monolayer models. Hence, unlike the 2D cell culture, the 3D cell culture in our biomimetic MC-B hydrogel matrix has the potential for innovative breakthroughs in the understanding of the role of ovarian CSCs in metastasis, chemotherapeutic resistance, and recurrence and in developing therapeutic strategies targeting ovarian CSCs. Further studies are needed to develop more useful and practical CSC-enrichment techniques to provide tools for a more comprehensive understanding of the essential features of ovarian CSC biology. These advances could yield valuable information for the development of CSC-directed therapy against OC, which could improve clinical outcomes.

## 4. Materials and Methods

### 4.1. Cell Culture

The ES-2 human OC cell line was purchased from the American Type Culture Collection (ATCC, Manassas, VA, USA). The A2780 cell line was obtained from the European Collection of Cell Cultures (ECACC, Salisbury, UK). R182, a chemotherapy-resistant human EOC cell line that originated from malignant ovarian ascites, was kindly donated by Dr. Jatin Shah (Memorial Sloan-Kettering Cancer Center, New York, NY, USA). All cell lines were cultured and maintained in RPMI 1640 (Hyclone, Chicago, IL, USA) supplemented with 10% fetal bovine serum (FBS, Welgene, Daegu, Korea) and 1% penicillin-streptomycin (Gibco/Thermo Fisher Scientific, Carlsbad, CA, USA) in a 5% CO_2_ humidified atmosphere at 37 °C. Subconfluent cells were harvested with trypsin-EDTA (Welgene) and used for further experiments. Media were replaced every third day.

### 4.2. Synthesis of Hydrogels for 3D Cell Culture

MC-B hydrogels for 3D cell culture were prepared as previously described by our group [37]. Briefly, sodium alginate was dissolved at 50 mg/mL in deionized water by constant stirring overnight at room temperature to prepare a 5% alginate stock solution and then autoclaved before use. To prepare the MC stock solution, MC powder was completely dissolved by vortexing and filtered in nuclease-free water at room temperature to make a 30% stock solution. An agarose stock solution (2%) was also made by adding high melting point agarose to deionized water, heating on a hot plate, and stirring occasionally until completely dissolved. A 565-μL volume of cells resuspended in culture medium (1 × 10^5^ cells/mL) were mixed with 240 μL of 5% sodium alginate solution in a 1.5-mL microcentrifuge tube at room temperature. This solution was then combined with 320 μL of 30% MC stock solution at room temperature to obtain 8% MC/1% alginate solution-containing cells. These cell suspensions were then blended carefully with 75 μL of 2% agarose solution at 35–40 °C to avoid cell damage. For the gelation of hydrogel solutions containing cells, the solutions were vortexed briefly, pipetted into 1-mL syringes, and finally incubated at 4 °C for 5–10 min. The gelled hydrogels were then transferred to the wells of 24-well plates containing 1.5 mL of culture medium and incubated at 37 °C. Media were changed every 2 days.

### 4.3. Spheroid Growth Assay

To evaluate the effects of MC-B hydrogels on the formation and growth of multicellular spheroids, A2780, ES-2, and R182 OC cells were cultured for desired times. The size of spheroids were measured at desired time points under a phase contrast microscope (IX70, Olympus, Tokyo, Japan). At least 20 spheroids on each hydrogel were photographed and their diameters measured. The diameter of a spheroid was defined as the average length of the diameters measured at two-degree intervals joining two outline points and passing through the centroid. The spheroid diameter was quantified and analyzed using an image analysis software (ImageJ, version 1.52a, National Institute of Health, Bethesda, MD, USA).

### 4.4. Cell Proliferation Assay

For the 2D culture, R182 cells were seeded at a density of approximately 1 × 10^4^ cells/well into 96-well plates, and for the 3D culture, the cells were seeded at a density of 1 × 10^5^ cells/mL. These cells were cultured in complete medium containing 10% FBS for 1, 3, 5, 8, 10, and 12 days. To measure cell proliferation, the WST-1 colorimetric assay was performed as per the manufacturer’s instructions (Daeil Lab Service, Seoul, Korea) in 96-well plates. In brief, plates were washed with phosphate-buffered saline (PBS). Ten microliters of WST-1 reagent was added to each well, and the plates were incubated for 1 h in a humidified chamber at 37 °C in 5% CO_2_. To quantify the metabolic cells, the formazan absorbance was measured at 450 nm by a microplate reader (Tecan, Männedorf, Switzerland). Cell viability was calculated as a percentage of the 2D control cell population. Cell morphology and spheroid size were also assessed at desired time points using a phase contrast microscope (IX70, Olympus). All experiments were independently performed at least three times.

### 4.5. Colony-Forming Assay

R182 cells cultured in 2D and 3D for 7 days were seeded into 6-well plates at a density of 200 cells/well. They were further grown for 3 days; at which time, suitable sized colonies were usually observed. Colonies were fixed with 100% methanol for 20 min at room temperature and washed with PBS. The colonies were subsequently stained with 0.5% crystal violet solution (Sigma-Aldrich, St Louis, MO, USA) for 5 min. After a second PBS wash, the plates were allowed to dry overnight. Stained colonies were counted to determine the number of colony-forming units. Each experiment was repeated three times.

### 4.6. Wound-Healing Assay

R182 cells cultured in 2D and 3D for 7 days were seeded at a density of 5 × 10^5^ cells/well in 6-well plates. When they attained complete confluence, the medium was changed to a starvation medium containing only 1% FBS. After making scratch wounds with a scratcher (SPL Life Sciences, Pocheon, Korea) in each well, the wells were rinsed with PBS to remove cellular debris and to avoid the reestablishment of displaced cells. The scratch closure was monitored and imaged at 0, 12, 24, 48, and 72 h using a phase contrast microscope. Each experiment was repeated three times.

### 4.7. Hydrogel Invasion Assay

A2780 cells cultured in 2D or 3D at 37 °C in a 5% CO_2_ humidified incubator for 7 days were seeded at a density of 5 × 10^5^ cells/well on the surface of freshly prepared MC-B hydrogels in 6-well plates. For confocal laser scanning microscopy analysis, the hydrogels containing 2D- and 3D-cultured cells were washed with PBS and then fixed for 20 min with cold 4% paraformaldehyde in 0.1-M phosphate buffer (pH 7.4). The fixative was removed by washing the hydrogels three times for 5 min each time with cold PBS, followed by permeabilization with 0.1% Triton X-100 in PBS for 5 min. After washing with cold PBS, the samples were blocked in 2% bovine serum albumin (Sigma-Aldrich) for 1 h at room temperature. Excess solution was removed, and hydrogels were incubated for 1 h at room temperature with 1:150 diluted FITC-phalloidin (Promega, Madison, WI, USA), rinsed in cold PBS, and mounted on glass slides using Vectashield^®^ containing 4′,6-diamidino-2-phenylindole (Vector Laboratories, Burlingame, CA, USA). Cell fluorescence was observed using a confocal laser scanning microscope (Olympus, Tokyo, Japan). To examine the depth of cell invasion, a stack of confocal images was collected using a step size of 5 μm. After thresholding, Z-stack images were used to generate reconstructed 3D projection images.

### 4.8. Extraction of RNA and Reverse Transcription-Polymerase Chain Reaction (RT-PCR)

Total RNA was isolated using the TRIzol reagent (Favorgen Biotech Corp, Pingtung, Taiwan) following the manufacturer’s recommendations. The RNA quantity and quality were assessed by measuring absorbance at 260 and 280 nm using a Nanodrop 2000 Spectrophotometer (Thermo Fisher Scientific, Waltham, MA, USA). Samples exhibiting an absorbance ratio (260/280) ≥ 1.9 were used. First-strand cDNA was synthesized by reverse transcription from 1 µg of total RNA in a 20-μL reaction mix (0.5-µg oligo (dT) 12–18 primers (Promega), 50-mM Tris-HCl (pH 8.3), 75-mM KCl, 3 mM MgCl_2_, 40 mM dithiothreitol, 0.5 mM deoxynucleotide triphosphate (dNTP; Promega), 10 U RNase inhibitors (Promega), and 200-U Moloney murine leukemia virus reverse transcriptase (Promega)). The reaction mix was incubated at 37 °C for 60 min and then heated to 70 °C for 5 min to stop the reaction. PCR amplification with gene-specific primers (sequences are shown in Table 1) was performed using an automated thermal cycler (Astec, Osaka, Japan). The obtained cDNA was added to the 20-μL PCR mix (20-mM Tris-HCl (pH 8.4), 50-mM KCl, 1.5-mM MgCl_2_, 0.1% Triton X-100, 0.2-mM dNTP (Promega), 0.5 pmol of each primer, and 5-U Taq DNA polymerase (Promega)) for use as a template. Once amplified, the DNA products were separated and detected using agarose gel electrophoresis (2% agarose gel stained with StaySafe™ Nucleic Acid Gel Stain; Real Biotech Corporation, Taipei, Taiwan) and photographed under ultraviolet light. The relative band intensities of the PCR products were measured using ImageJ software. The results were expressed as ratios vs. a housekeeping gene (glyceraldehyde-3-phosphate dehydrogenase, GAPDH) amplified from the same cDNA samples.

### 4.9. Western Blot Analysis

After R182 cells were cultured in 2D and 3D for 7 days, they were harvested and washed twice with ice-cold PBS. These were then lysed in RIPA lysis buffer (GenDEPOT, Barker, TX, USA) containing a protease inhibitor cocktail (GenDEPOT) for 30 min (for 2D-cultured cells) and 3 h (for 3D-cultured cells) on ice with agitation by vortexing at 10 min intervals. The resulting homogenates were centrifuged at 17,900× *g* for 30 min at 4 °C, and the supernatants were collected. Protein concentration was determined using the bicinchoninic acid (BCA) protein assay (Sigma-Aldrich). An equal amount of protein per sample was mixed with Laemmli sample buffer (Bio-Rad, Hercules, CA, USA) and loaded (30 μg/lane) onto a 12% (*v/v*) sodium dodecyl sulfate-polyacrylamide gel for electrophoresis. Separated protein was blotted onto a polyvinylidene fluoride membrane (Amersham Biosciences, Piscataway, NJ, USA) via the semi-dry transfer method (Bio-Rad). The membranes were blocked with 3% BSA in Tris-buffered saline with 0.1% Tween 20 at room temperature for 1 h. The membranes were washed and incubated overnight at 4 °C with primary polyclonal antibodies against Bax (1:2000, Santa Cruz Biotechnology, Dallas, TX, USA) and Bcl-2 (1:2000, Santa Cruz Biotechnology), and subsequently incubated for 1 h at room temperature with anti-mouse polyclonal secondary antibody (1:10,000, Cell Signaling Technology, Danvers, MA, USA). Following the wash steps, the proteins were visualized using the enhanced chemiluminescence (ECL) kit (Amersham Biosciences) according to the manufacturer’s instructions. Images were captured by the Amersham Imager 680 (Amersham Biosciences) and quantified with the ImageJ software.

### 4.10. Flow Cytometry

To detect the CSC population, A2780, ES-2, and R182 cells were cultured in 2D and 3D for 7 days and were harvested by pipetting. They were washed with Hanks’ balanced salt solution (HBSS, Gibco/Thermo Fisher Scientific) containing 0.1% BSA and 0.1% sodium azide and filtered through a 100-μm cell strainer (SPL Life Sciences). Phenotypic analysis of cell surface marker expression was performed by flow cytometry. Briefly, cells were washed twice with HBSS and resuspended in the cell-staining buffer. Cells were immunostained for cell surface markers by incubating them for 30 min with phycoerythrin (PE)-Cy7-labeled anti-CD44 (1:10, Invitrogen, Life Technologies, Carlsbad, CA, USA), PE-labeled anti-CD117 (1:10, BioLegend, San Diego, CA, USA), and allophycocyanin-labeled anti-CD133 (1:10, BioLegend) monoclonal antibodies. For control, 2D-cultured cells were used. Fluorescence-activated cell sorting analysis was performed using a FACS Canto-II flow cytometer (BD Biosciences, San Jose, CA, USA). Flow cytometry data were analyzed using FlowJo 10.3.0 (Tree Star, Ashland, OR, USA).

### 4.11. Chemotherapeutic Sensitivity Assay

After R182 cells were cultured in 2D and 3D for 7 days, the cell culture media were replaced with serum-free media containing 12-μM cisplatin (Sigma-Aldrich), 100-nM docetaxel (Sigma-Aldrich), and 1-μM doxorubicin (Cell Signaling Technology) for 48 h. To determine cell viability, a WST-1 assay was performed as previously described. All experiments were performed in triplicate and repeated three times.

### 4.12. Statistical Analysis

All quantitative results are expressed as mean ± standard deviation of at least three independent experiments. Comparisons between two groups were analyzed by Student’s *t*-test. Values of * *p* < 0.05, ** *p* < 0.01, and *** *p* < 0.001 were considered statistically significant.

## 5. Conclusions

We present an effective 3D in vitro culture method based on our MC-B hydrogel matrix developed and optimized for growing multicellular OC spheres derived from different OC cell lines. We demonstrated its usefulness in the isolation and enrichment of ovarian CSCs. The 3D in vitro OC cell culture using the MC-B hydrogel scaffold offers several advantages. These include simplicity, reproducibility, bioactivity, efficiency, and low cost. OC cells grown in the 3D culture system exhibited biochemical and physiological features. These included (1) enhanced cell proliferation, migration, and invasion, as well as colony formation and chemotherapy resistance; (2) suppressed apoptosis; (3) the upregulated expression of multidrug resistance-related genes (MDR1 and MRP1); (4) elevated levels of key molecules associated with tumor progression and malignancy, such as apoptosis-regulating molecules (Bcl-2 and Bax); EMT transcription factors (Snail, Slug, and Twist); Notch (Notch-1 and -2); tumor stemness and pluripotency biomarkers (Sox2, Oct4, Nanog, and KLF4); and (5) the enrichment of an ovarian CSC population by approximately up to 56-fold compared to the 2D-based conventional suspension cell culture. The 3D in vitro OC model is a promising in vitro research platform to study OC and ovarian CSC biology, as well as to screen new anti-OC and anti-ovarian CSC-targeted therapeutics.

## Figures and Tables

**Table 1:** Reverse transcription (RT-PCR) primer names and their sequences.

Gene Name	Forward (5′-3′)	Reverse (5′-3′)
KLF4	GGCACTACCGTAAACACACG	CTGGCAGTGTGGGTCATATC
MDR1	GAGCCTACTTGGTGGCACAT	TCCTTCCAATGTGTTCGGCA
MRP1	AATGCGCCAAGACTAGGAAG	ACCGGAGGATGTTGAACAAG
Nanog	GTCTTCTGCTGAGATGCCTCACA	CTTCTGCGTCACACCATTGCTAT
Notch-1	TACAAGTGCGACTGTGACCC	CACACGTAGCCACTGGTCAT
Notch-2	CAACCGCAATGGAGGCTATG	GCGAAGGCACAATCATCAATGTT
Oct4	ATCCTGGGGGTTCTATTTGG	TCTCCAGGTTGCCTCTCACT
Slug	GGTCAAGAAGCATTTCAAC	GGTAATGTGTGGGTCCGA
Snail	AGACCCACTCAGATGTCAA	CATAGTTAGTCACACCTCGT
Sox2	AACCAGCGCATGGACAGTTA	GACTTGACCACCGAACCCAT
Twist	GTCCGCAGTCTTACGAGGAG	GCTTGAGGGTCTGAATCTTGCT
GAPDH	AAGTGGATATTGTTGCCATC	ACTGTGGTCATGAGTCCTTC

## References

[1] Siegel R., Miller K.D., Jemal A. (2018). Cancer statistics, 2018. CA Cancer J. Clin..

[2] Torre L.A., Trabert B., DeSantis C.E., Miller K.D., Samimi G., Runowicz C.D., Gaudet M.M., Jemal A., Siegel R.L. (2018). Ovarian cancer statistics, 2018. CA Cancer J. Clin..

[3] Ghoneum A., Afify H., Salih Z., Kelly M., Said N. (2018). Role of tumor microenvironment in the pathobiology of ovarian cancer: Insights and therapeutic opportunities. Cancer Med..

[4] Reid B.M., Permuth J.B., Sellers T.A. (2017). Epidemiology of ovarian cancer: A review. Cancer Biol. Med..

[5] Pokhriyal R., Hariprasad R., Kumar L., Hariprasad G. (2019). Chemotherapy Resistance in Advanced Ovarian Cancer Patients. Biomark. Cancer.

[6] Bapat S.A., Mali A.M., Koppikar C.B., Kurrey N.K. (2005). Stem and progenitor-like cells contribute to the aggressive behavior of human epithelial ovarian cancer. Cancer Res..

[7] Singh A., Settleman J. (2010). EMT, cancer stem cells and drug resistance: An emerging axis of evil in the war on cancer. Oncogene.

[8] Abdullah L.N., Chow E.K. (2013). Mechanisms of chemoresistance in cancer stem cells. Clin. Transl. Med..

[9] Bai X., Ni J., Beretov J., Graham P., Li Y. (2018). Cancer stem cell in breast cancer therapeutic resistance. Cancer Treat. Rev..

[10] Shibue T., Weinberg R.A. (2017). EMT, CSCs, and drug resistance: The mechanistic link and clinical implications. Nat. Rev. Clin. Oncol..

[11] Li F., Tiede B., Massagué J., Kang Y. (2007). Beyond tumorigenesis: Cancer stem cells in metastasis. Cell Res..

[12] Dieter S.M., Ball C.R., Hoffmann C.M., Nowrouzi A., Herbst F., Zavidij O., Abel U., Arens A., Weichert W., Brand K. (2011). Distinct types of tumor-initiating cells from human colon cancer tumors and metastases. Cell Stem Cell.

[13] Steffensen K.D., Alvero A.B., Yang Y., Waldstrom M., Hui P., Holmberg J.C., Silasi D.A., Jakobsen A., Rutherford T., Mor G. (2011). Prevalence of epithelial ovarian cancer stem cells correlates with recurrence in early-stage ovarian cancer. J. Oncol..

[14] Lawson D.A., Bhakta N.R., Kessenbrock K., Prummel K.D., Yu Y., Takai K., Zhou A., Eyob H., Balakrishnan S., Wang C.-Y. (2015). Single-cell analysis reveals a stem-cell program in human metastatic breast cancer cells. Nature.

[15] Aponte P.M., Caicedo A. (2017). Stemness in Cancer: Stem Cells, Cancer Stem Cells, and Their Microenvironment. Stem Cells Int..

[16] Lathia J., Liu H., Matei D. (2020). The Clinical Impact of Cancer Stem Cells. Oncologist.

[17] Keyvani V., Farshchian M., Esmaeili S.A., Yari H., Moghbeli M., Nezhad S.K., Abbaszadegan M.R. (2019). Ovarian cancer stem cells and targeted therapy. J. Ovarian Res..

[18] Zong X., Nephew K.P. (2019). Ovarian Cancer Stem Cells: Role in Metastasis and Opportunity for Therapeutic Targeting. Cancers.

[19] Liang Z.M., Chen Y., Luo M.L. (2017). Targeting Stemness: Implications for Precision Medicine in Breast Cancer. Adv. Exp. Med. Biol..

[20] Phi L.T.H., Sari I.N., Yang Y.G., Lee S.H., Jun N., Kim K.S., Lee Y.K., Kwon H.Y. (2018). Cancer Stem Cells (CSCs) in Drug Resistance and their Therapeutic Implications in Cancer Treatment. Stem Cells Int..

[21] Al-Alem L.F., Pandya U.M., Baker A.T., Bellio C., Zarrella B.D., Clark J., DiGloria C.M., Rueda B.R. (2019). Ovarian cancer stem cells: What progress have we made?. Int. J. Biochem. Cell Biol..

[22] Bhaskara V.K., Mohanam I., Rao J.S., Mohanam S. (2012). Intermittent hypoxia regulates stem-like characteristics and differentiation of neuroblastoma cells. PLoS ONE.

[23] Hu X., Ghisolfi L., Keates A.C., Zhang J., Xiang S., Lee D.K., Li C.J. (2012). Induction of cancer cell stemness by chemotherapy. Cell Cycle.

[24] Yang L., Lai D. (2013). Ovarian cancer stem cells enrichment. Methods Mol. Biol..

[25] Bielecka Z.F., Maliszewska-Olejniczak K., Safir I.J., Szczylik C., Czarnecka A.M. (2017). Three-dimensional cell culture model utilization in cancer stem cell research. Biol. Rev. Camb. Philos. Soc..

[26] Katt M.E., Placone A.L., Wong A.D., Xu Z.S., Searson P.C. (2016). In Vitro Tumor Models: Advantages, Disadvantages, Variables, and Selecting the Right Platform. Front. Bioeng. Biotechnol..

[27] Jensen C., Teng Y. (2020). Is It Time to Start Transitioning from 2D to 3D Cell Culture?. Front. Mol. Biosci..

[28] Gorgieva S., Kokol V. (2011). Biomaterials Applications for Nanomedicine.

[29] Kular J.K., Basu S., Sharma R.I. (2014). The extracellular matrix: Structure, composition, age-related differences, tools for analysis and applications for tissue engineering. J. Tissue Eng..

[30] Brinckmann J. (2005). Collagens at a Glance. Top. Curr. Chem..

[31] Lee C.H., Singla A., Lee Y. (2001). Biomedical applications of collagen. Int. J. Pharm..

[32] Colchester A.C., Colchester N.T. (2005). The origin of bovine spongiform encephalopathy: The human prion disease hypothesis. Lancet.

[33] Easterbrook C., Maddern G. (2008). Porcine and bovine surgical products: Jewish, Muslim, and Hindu perspectives. Arch. Surg..

[34] Yamada S., Yamamoto K., Ikeda T., Yanagiguchi K., Hayashi Y. (2014). Potency of fish collagen as a scaffold for regenerative medicine. Biomed. Res. Int..

[35] Yamamoto K., Igawa K., Sugimoto K., Yoshizawa Y., Yanagiguchi K., Ikeda T., Yamada S., Hayashi Y. (2014). Biological safety of fish (tilapia) collagen. Biomed. Res. Int..

[36] Lim Y.S., Ok Y.J., Hwang S.Y., Kwak J.Y., Yoon S. (2019). Marine Collagen as A Promising Biomaterial for Biomedical Applications. Mar. Drugs..

[37] Shin S., Ikram M., Subhan F., Kang H.Y., Lim Y., Lee R., Jin S., Jeong Y.H., Kwak J.Y., Na Y.J. (2016). Alginate–marine collagen–agarose composite hydrogels as matrices for biomimetic 3D cell spheroid formation. RSC Adv..

[38] Ikram M., Lim Y., Baek S.Y., Jin S., Jeong Y.H., Kwak J.Y., Yoon S. (2017). Co-targeting of Tiam1/Rac1 and Notch ameliorates chemoresistance against doxorubicin in a biomimetic 3D lymphoma model. Oncotarget.

[39] Dudas J., Ladanyi A., Ingruber J., Steinbichler T.B., Riechelmann H. (2020). Epithelial to Mesenchymal Transition: A Mechanism that Fuels Cancer Radio/Chemoresistance. Cells.

[40] Ribatti D., Tamma R., Annese T. (2020). Epithelial-Mesenchymal Transition in Cancer: A Historical Overview. Transl. Oncol..

[41] Artacho-Cordon A., Artacho-Cordon F., Rios-Arrabal S., Calvente I., Nunez M.I. (2012). Tumor microenvironment and breast cancer progression: A complex scenario. Cancer Biol. Ther..

[42] Arvelo F., Sojo F., Cotte C. (2016). Tumour progression and metastasis. Ecancermedicalscience.

[43] Kim J.B., Stein R., O’Hare M.J. (2004). Three-dimensional in vitro tissue culture models of breast cancer—A review. Breast Cancer Res. Treat..

[44] Ocana A., Pandiella A., Siu L.L., Tannock I.F. (2010). Preclinical development of molecular-targeted agents for cancer. Nat. Rev. Clin. Oncol..

[45] Hutchinson L., Kirk R. (2011). High drug attrition rates—Where are we going wrong?. Nat. Rev. Clin. Oncol..

[46] Herter-Sprie G.S., Kung A.L., Wong K.K. (2013). New cast for a new era: Preclinical cancer drug development revisited. J. Clin. Investig..

[47] Fong M.Y., Kakar S.S. (2009). Ovarian cancer mouse models: A summary of current models and their limitations. J. Ovarian Res..

[48] Bobbs A.S., Cole J.M., Dahl K.D.C. (2015). Emerging and Evolving Ovarian Cancer Animal Models. Cancer Growth Metastasis.

[49] Hasan N., Ohman A.W., Dinulescu D.M. (2015). The promise and challenge of ovarian cancer models. Transl. Cancer Res..

[50] Decaup E., Jean C., Laurent C., Gravelle P., Fruchon S., Capilla F., Marrot A., Al Saati T., Frenois F.X., Laurent G. (2013). Anti-tumor activity of obinutuzumab and rituximab in a follicular lymphoma 3D model. Blood Cancer J..

[51] Szakács G., Paterson J.K., Ludwig J.A., Booth-Genthe C., Gottesman M.M. (2006). Targeting multidrug resistance in cancer. Nat. Rev. Drug Discov..

[52] Barker H.E., Paget J.T., Khan A.A., Harrington K.J. (2015). The tumour microenvironment after radiotherapy: Mechanisms of resistance and recurrence. Nat. Rev. Cancer.

[53] Jo Y., Choi N., Kim K., Koo H.J., Choi J., Kim H.N. (2018). Chemoresistance of Cancer Cells: Requirements of Tumor Microenvironment-mimicking in Vitro Models in Anti-Cancer Drug Development. Theranostics.

[54] Choi Y.H., Yu A.M. (2014). ABC transporters in multidrug resistance and pharmacokinetics, and strategies for drug development. Curr. Pharm. Des..

[55] Wind N.S., Holen I. (2011). Multidrug resistance in breast cancer: From in vitro models to clinical studies. Int. J. Breast Cancer.

[56] Enmon R., Yang W.H., Ballangrud A.M., Solit D.B., Heller G., Rosen N., Scher H.I., Sgouros G. (2003). Combination treatment with 17-N-allylamino-17-demethoxy geldanamycin and acute irradiation produces supra-additive growth suppression in human prostate carcinoma spheroids. Cancer Res..

[57] Lambert B., De Ridder L., Slegers G., De Gelder V., Dierckx R.A., Thierens H. (2004). Screening for supra-additive effects of cytotoxic drugs and gamma irradiation in an in vitro model for hepatocellular carcinoma. Can. J. Physiol. Pharmacol..

[58] Friedrich J., Seidel C., Ebner R., Kunz-Schughart L.A. (2009). Spheroid-based drug screen: Considerations and practical approach. Nat. Protoc..

[59] Vinci M., Gowan S., Boxall F., Patterson L., Zimmermann M., Court W., Lomas C., Mendiola M., Hardisson D., Eccles S.A. (2012). Advances in establishment and analysis of three-dimensional tumor spheroid-based functional assays for target validation and drug evaluation. BMC Biol..

[60] Zanoni M., Piccinini F., Arienti C., Zamagni A., Santi S., Polico R., Bevilacqua A., Tesei A. (2016). 3D tumor spheroid models for in vitro therapeutic screening: A systematic approach to enhance the biological relevance of data obtained. Sci. Rep..

[61] Brown J.M. (2007). Tumor hypoxia in cancer therapy. Methods Enzymol..

[62] Milane L., Ganesh S., Shah S., Duan Z.F., Amiji M. (2011). Multi-modal strategies for overcoming tumor drug resistance: Hypoxia, the Warburg effect, stem cells, and multifunctional nanotechnology. J. Control. Release.

[63] Liang S., Galluzzo P., Sobol A., Skucha S., Rambo B., Bocchetta M. (2012). Multimodality Approaches to Treat Hypoxic Non-Small Cell Lung Cancer (NSCLC) Microenvironment. Genes Cancer.

[64] Chen J., Ding Z., Peng Y., Pan F., Li J., Zou L., Zhang Y., Liang H. (2014). HIF-1α inhibition reverses multidrug resistance in colon cancer cells via downregulation of MDR1/P-glycoprotein. PLoS ONE.

[65] Arnold C.R., Mangesius J., Skvortsova I.I., Ganswindt U. (2020). The Role of Cancer Stem Cells in Radiation Resistance. Front. Oncol..

[66] Nguyen L.V., Vanner R., Dirks P., Eaves C.J. (2012). Cancer stem cells: An evolving concept. Nat. Rev. Cancer.

[67] Chen K., Huang Y.H., Chen J.L. (2013). Understanding and targeting cancer stem cells: Therapeutic implications and challenges. Acta Pharmacol. Sin..

[68] Prieto-Vila M., Takahashi R.U., Usuba W., Kohama I., Ochiya T. (2017). Drug Resistance Driven by Cancer Stem Cells and Their Niche. Int. J. Mol. Sci..

[69] Atashzar M.R., Baharlou R., Karami J., Abdollahi H., Rezaei R., Pourramezan F., Zoljalali Moghaddam S.H. (2019). Cancer stem cells: A review from origin to therapeutic implications. J. Cell. Physiol..

[70] Zhao J. (2016). Cancer stem cells and chemoresistance: The smartest survives the raid. Pharmacol. Ther..

[71] Nedeljkovic M., Damjanivic A. (2019). Mechanisms of Chemotherapy Resistance in Triple-Negative Breast Cancer—How We Can Rise to the Challenge. Cells.

[72] Barbato L., Bocchetti M., Di Biase A., Regad T. (2019). Cancer Stem Cells and Targeting Strategies. Cells.

[73] Liu W.H., Wang X., You N., Tao K.S., Wang T., Tang L.J., Dou K.F. (2012). Efficient enrichment of hepatic cancer stem-like cells from a primary rat HCC model via a density gradient centrifugation-centered method. PLoS ONE.

[74] O’Reilly D., Johnson P., Buchanan P.J. (2019). Hypoxia induced cancer stem cell enrichment promotes resistance to androgen deprivation therapy in prostate cancer. Steroids.

[75] Lu H., Chen I., Shimoda L.A., Park Y., Zhang C., Tran L., Zhang H., Semenza G.L. (2017). Chemotherapy-Induced Ca^2+^ Release Stimulates Breast Cancer Stem Cell Enrichment. Cell Rep..

[76] Jiang P., Xu C., Zhou M., Zhou H., Dong W., Wu X., Chen A., Feng Q. (2018). RXRα-enriched cancer stem cell-like properties triggered by CDDP in head and neck squamous cell carcinoma (HNSCC). Carcinogenesis.

[77] Zhou B., Jin Y., Zhang D., Lin D. (2018). 5-Fluorouracil may enrich cancer stem cells in canine mammary tumor cells in vitro. Oncol. Lett..

[78] Chien C.Y., Chuang H.C., Chen C.H. (2012). The side population of cancer stem-like cells in human oral cancer. Oral Oncol..

[79] Yasuda K., Torigoe T., Morita R., Kuroda T., Takahashi A., Matsuzaki J., Kochin V., Asanuma H., Hasegawa T., Saito T. (2013). Ovarian cancer stem cells are enriched in side population and aldehyde dehydrogenase bright overlapping population. PLoS ONE.

[80] Jiménez G., Hackenberg M., Catalina P., Boulaiz H., Griñán-Lisón C., García M.Á., Perán M., López-Ruiz E., Ramírez A., Morata-Tarifa C. (2018). Mesenchymal stem cell’s secretome promotes selective enrichment of cancer stem-like cells with specific cytogenetic profile. Cancer Lett..

[81] Yaiza J.M., Gloria R.A., María Belén G.O., Elena L.R., Gema J., Juan Antonio M., María Ángel G.C., Houria B. (2019). Melanoma cancer stem-like cells: Optimization method for culture, enrichment and maintenance. Tissue Cell.

[82] Watanabe Y., Yoshimura K., Yoshikawa K., Tsunedomi R., Shindo Y., Matsukuma S., Maeda N., Kanekiyo S., Suzuki N., Kuramasu A. (2014). A stem cell medium containing neural stimulating factor induces a pancreatic cancer stem-like cell-enriched population. Int. J. Oncol..

[83] Weitzenfeld P., Meshel T., Ben-Baruch A. (2016). Microenvironmental networks promote tumor heterogeneity and enrich for metastatic cancer stem-like cells in Luminal-A breast tumor cells. Oncotarget.

[84] Goyette S., Liang Y., Mafuvadze B., Cook M.T., Munir M., Hyder S.M. (2017). Natural and synthetic progestins enrich cancer stem cell-like cells in hormone-responsive human breast cancer cell populations in vitro. Breast Cancer.

[85] Lu H., Tran L., Park Y., Chen I., Lan J., Xie Y., Semenza G.L. (2018). Reciprocal Regulation of DUSP9 and DUSP16 Expression by HIF1 Controls ERK and p38 MAP Kinase Activity and Mediates Chemotherapy-Induced Breast Cancer Stem Cell Enrichment. Cancer Res..

[86] Saltanatpour Z., Johari B., Alizadeh A., Lotfinia M., Majidzadeh-A K., Nikbin B., Kadivar M. (2019). Enrichment of cancer stem-like cells by the induction of epithelial-mesenchymal transition using lentiviral vector carrying E-cadherin shRNA in HT29 cell line. J. Cell. Physiol..

[87] Abbasian M., Mousavi E., Khalili M., Arab-Bafrani Z. (2019). Using of keratin substrate for enrichment of HT29 colorectal cancer stem-like cells. J. Biomed. Mater. Res. B Appl. Biomater..

[88] Bellio C., DiGloria C., Foster R., James K., Konstantinopoulos P.A., Growdon W.B., Rueda B.R. (2019). PARP Inhibition Induces Enrichment of DNA Repair-Proficient CD133 and CD117 Positive Ovarian Cancer Stem Cells. Mol. Cancer Res..

[89] Yang J., Yang L., Li S., Hu N. (2020). HGF/c-Met Promote Renal Carcinoma Cancer Stem Cells Enrichment through Upregulation of Cir-CCDC66. Technol. Cancer Res. Treat..

[90] McKee C., Chaudhry G.R. (2017). Advances and challenges in stem cell culture. Colloids Surf. B Biointerfaces.

[91] Sart S., Tsai A.C., Li Y., Ma T. (2014). Three-dimensional aggregates of mesenchymal stem cells: Cellular mechanisms, biological properties, and applications. Tissue Eng. Part B Rev..

[92] Boo L., Ho W.Y., Ali N.M., Yeap S.K., Ky H., Chan K.G., Yin W.F., Satharasinghe D.A., Liew W.C., Tan S.W. (2016). MiRNA Transcriptome Profiling of Spheroid-Enriched Cells with Cancer Stem Cell Properties in Human Breast MCF-7 Cell Line. Int. J. Biol. Sci..

[93] Van de Walle A., Wilhelm C., Luciani N. (2017). 3D Magnetic Stem Cell Aggregation and Bioreactor Maturation for Cartilage Regeneration. J. Vis. Exp..

[94] Lee J.W., Sung J.S., Park Y.S., Chung S., Kim Y.H. (2018). Isolation of spheroid-forming single cells from gastric cancer cell lines: Enrichment of cancer stem-like cells. Biotechniques.

[95] Balla M.M.S., Yadav H.D., Pandey B.N. (2019). Tumorsphere assay provides a better in vitro method for cancer stem-like cells enrichment in A549 lung adenocarcinoma cells. Tissue Cell.

[96] Gao W., Wu D., Wang Y., Wang Z., Zou C., Dai Y., Ng C.F., Teoh J.Y., Chan F.L. (2018). Development of a novel and economical agar-based non-adherent three-dimensional culture method for enrichment of cancer stem-like cells. Stem Cell Res. Ther..

[97] Herheliuk T., Perepelytsina O., Ugnivenko A., Ostapchenko L., Sydorenko M. (2019). Investigation of multicellular tumor spheroids enriched for a cancer stem cell phenotype. Stem Cell Investig..

[98] Ma X.L., Sun Y.F., Wang B.L., Shen M.N., Zhou Y., Chen J.W., Hu B., Gong Z.J., Zhang X., Cao Y. (2019). Sphere-forming culture enriches liver cancer stem cells and reveals Stearoyl-CoA desaturase 1 as a potential therapeutic target. BMC Cancer.

[99] Ward Rashidi M.R., Mehta P., Bregenzer M., Raghavan S., Fleck E.M., Horst E.N., Harissa Z., Ravikumar V., Brady S., Bild A. (2019). Engineered 3D Model of Cancer Stem Cell Enrichment and Chemoresistance. Neoplasia.

[100] Zweigerdt R., Olmer R., Singh H., Haverich A., Martin U. (2011). Scalable expansion of human pluripotent stem cells in suspension culture. Nat. Protoc..

[101] Gong X., Lin C., Cheng J., Su J., Zhao H., Liu T., Wen X., Zhao P. (2015). Generation of Multicellular Tumor Spheroids with Microwell-Based Agarose Scaffolds for Drug Testing. PLoS ONE.

[102] Abe-Fukasawa N., Otsuka K., Aihara A., Itasaki N., Nishino T. (2018). Novel 3D Liquid Cell Culture Method for Anchorage-independent Cell Growth, Cell Imaging and Automated Drug Screening. Sci. Rep..

[103] Zhu Z.W., Chen L., Liu J.X., Huang J.W., Wu G., Zheng Y.F., Yao K.T. (2018). A novel three-dimensional tumorsphere culture system for the efficient and low-cost enrichment of cancer stem cells with natural polymers. Exp. Ther. Med..

[104] Kievit F.M., Florczyk S.J., Leung M.C., Wang K., Wu J.D., Silber J.R., Ellenbogen R.G., Lee J.S., Zhang M. (2014). Proliferation and enrichment of CD133(+) glioblastoma cancer stem cells on 3D chitosan-alginate scaffolds. Biomaterials.

[105] Lee I.C., Chang J.F. (2015). Label-free selection and enrichment of liver cancer stem cells by surface niches build up with polyelectrolyte multilayer films. Colloids Surf. B Biointerfaces.

[106] Lee I.C., Chuang C.C., Wu Y.C. (2015). Niche Mimicking for Selection and Enrichment of Liver Cancer Stem Cells by Hyaluronic Acid-Based Multilayer Films. ACS Appl. Mater. Interfaces.

[107] Florczyk S.J., Kievit F.M., Wang K., Erickson A.E., Ellenbogen R.G., Zhang M. (2016). 3D Porous Chitosan-Alginate Scaffolds Promote Proliferation and Enrichment of Cancer Stem-Like Cells. J. Mater. Chem. B.

[108] Palomeras S., Rabionet M., Ferrer I., Sarrats A., Garcia-Romeu M.L., Puig T., Ciurana J. (2016). Breast Cancer Stem Cell Culture and Enrichment Using Poly(ε-Caprolactone) Scaffolds. Molecules.

[109] Bahmad H.F., Cheaito K., Chalhoub R.M., Hadadeh O., Monzer A., Ballout F., El-Hajj A., Mukherji D., Liu Y.N., Daoud G. (2018). Sphere-Formation Assay: Three-Dimensional in vitro Culturing of Prostate Cancer Stem/Progenitor Sphere-Forming Cells. Front. Oncol..

[110] Guo X., Chen Y., Ji W., Chen X., Li C., Ge R. (2019). Enrichment of cancer stem cells by agarose multi-well dishes and 3D spheroid culture. Cell Tissue Res..

[111] Tan S., Yamashita A., Gao S.J., Kurisawa M. (2019). Hyaluronic acid hydrogels with defined crosslink density for the efficient enrichment of breast cancer stem cells. Acta Biomater..

[112] Tosello V., Ferrando A.A. (2013). The NOTCH signaling pathway: Role in the pathogenesis of T-cell acute lymphoblastic leukemia and implication for therapy. Ther. Adv. Hematol..

[113] Wang Z., Li Y., Ahmad A., Azmi A.S., Banerjee S., Kong D., Sarkar F.H. (2010). Targeting Notch signaling pathway to overcome drug resistance for cancer therapy. Biochim. Biophys. Acta.

[114] Cho S., Lu M., He X., Ee P.L.R., Bhat U., Schneider E., Miele L., Beck W.T. (2011). Notch1 regulates the expression of the multidrug resistance gene ABCC1/MRP1 in cultured cancer cells. Proc. Natl. Acad. Sci. USA.

[115] Huang J., Chen Y., Li J., Zhang K., Chen J., Chen D., Feng B., Song H., Feng J., Wang R. (2016). Notch-1 Confers Chemoresistance in Lung Adenocarcinoma to Taxanes through AP-1/microRNA-451 Mediated Regulation of MDR-1. Mol. Ther. Nucleic Acids.

